# The NIH 2025 Public Access Policy: Immediate access, unequal costs

**DOI:** 10.1371/journal.pmed.1005124

**Published:** 2026-06-01

**Authors:** Caitlin R. Ryus, Caroline Raymond King, Edward R. Melnick

**Affiliations:** 1 Department of Emergency Medicine, Yale School of Medicine, New Haven, Connecticut, United States of America; 2 Department of Biostatistics (Health Informatics), Yale School of Public Health, New Haven, Connecticut, United States of America

## Abstract

In this Perspective, Caitlin Ryus and colleagues discuss the NIH 2025 Public Access Policy, highlighting that while expanding who can read science, the policy risks creating new barriers to who can afford to perform and publish their science.

## Teaser

The NIH 2025 Public Access Policy eliminates embargo periods for federally funded research, expanding who can read science. Yet without addressing article processing charges and market concentration, the policy risks creating new barriers to who can afford to perform and publish their science.

## Introduction

In 2025, the National Institutes of Health (NIH) completed implementation of its updated Public Access Policy, requiring that all NIH-funded research articles be made immediately available upon publication, eliminating the 12-month embargo period previously permitted [1]. This policy fulfills a principle articulated by the open science movement for more than two decades: that the results of publicly funded research should be promptly and freely accessible to scientists, clinicians, patients, and the public.

Public access—the free availability of publicly funded research outputs—is a central goal of the broader open science movement. Open access publishing, in which the final version of record is freely available (often through article processing charges (APCs)), is one mechanism for achieving this goal, but the two are not equivalent.

Unfortunately, an unsolved problem remains: who pays for freely available peer-reviewed research results? This Perspective examines how the NIH policy intersects with contemporary publishing economics. We argue that while public access has expanded who can read science, less attention has been paid to who can afford to produce and publish it, and how the labor sustaining scientific communication is governed.

## Scientific labor and publishing economics

The modern open access movement emerged in response to concerns that escalating journal subscription prices were restricting access to scientific literature, even within well-resourced academic institutions. As digital publishing reduced technical barriers to dissemination, economic barriers persisted. Over the subsequent decades, open access expanded rapidly, culminating in the NIH policy eliminating embargo periods [2].

In many open access models, costs have shifted from readers to researchers through APCs. In many selective journals, APCs now routinely exceed $3,000 and may reach upwards of $12,000 [3]. These charges have become a central component of publisher revenue.

Academic publishing remains highly concentrated among a small number of commercial publishers [4] whose profit margins approach 40%, rivaling the most profitable corporations [5,6]. By contrast, nonprofit models operate at substantially lower-cost; for example, per-article download costs have been estimated to be as low as $0.04 for nonprofit publications compared with $1.06 in some for-profit journals under large institutional contracts [7]. Although publishers provide essential services including infrastructure, production, and archiving, the gap between production costs and prices raises questions about how fees are set and revenues distributed [5,6]. When publication fees are charged to federally funded grants, the public may effectively pay multiple times—through direct research funding, publication costs, and institutional overhead (indirect costs).

Scientific publishing depends on extensive expert labor: researchers conduct studies and prepare manuscripts, while peer reviewers and academic editors evaluate work and safeguard the scientific record, largely without compensation. This arrangement inverts the logic of most content industries. In book publishing, for example, publishers pay authors and editors, then charge readers modest prices. In academic publishing, authors pay to publish, reviewers work without compensation, and readers pay substantial fees to access the results—often for a single article. Although the academic community governs what enters the literature, it has little influence over pricing, access models, or publishing contracts [6]. As a result, unpaid scholarly labor underpins a system in which financial returns accrue elsewhere, and control over dissemination is frequently transferred away from the scientists and institutions that produced the work.

## The NIH 2025 policy: achievement and constraint

The NIH Public Access Policy addresses a core ethical principle: the public should have timely access to the results of research it funds [1]. The policy strengthens accessibility and accelerates scientific exchange, aligning US policy with global open science initiatives.

Although compliance with the NIH Public Access Policy can be achieved without APCs by depositing the author-accepted manuscript (AAM) in PubMed Central, this zero-cost pathway is only available if the journal permits immediate AAM deposit. Some major commercial publishers have moved to eliminate this option, leaving APC-funded open access as the only compliant pathway for authors publishing in their journals. Other publishers have begun conditioning their own depositing of the final published article in PubMed Central on payment of APCs, despite lacking the authority to restrict authors from depositing their accepted manuscripts [2].

A mechanism already exists to address these constraints: the Federal Purpose License. Under federal grant terms, the US government retains a royalty-free, irrevocable right to reproduce and distribute work produced under its awards. This provision supports authors’ ability to deposit accepted manuscripts in PubMed Central regardless of publisher policies. Yet in practice, researchers often remain responsible for navigating conflicting requirements and absorbing associated costs.

The policy does not address APC pricing, market concentration, or the incentives that shape publication decisions. Although APCs may be allowable grant expenses, funders do not negotiate prices or assess whether fees reflect underlying costs or public value.

Implementation also reflects an asymmetry in enforcement. Noncompliant articles may be flagged in NIH reporting systems, placing responsibility on authors who may have limited control over journal policies or insufficient resources to pay APCs. By contrast, publishers face few direct consequences when compliance pathways are costly or unclear.

In a prestige-driven academic environment, researchers often perceive limited freedom to select lower-cost venues without career risk. Metrics such as journal impact factor and reputation continue to shape hiring, promotion, and funding decisions [8]. Consequently, immediate access mandates may increase reliance on APC-funded publishing while leaving existing pricing power largely intact. Funder-imposed APC caps, while appealing as a near-term intervention, risk functioning as a market floor rather than a ceiling, potentially reducing price competition and deepening inequities between well- and under-resourced institutions and investigators [9]. Structural alternatives, including asserting the Federal Purpose License and investing in nonprofit and community-owned publishing infrastructure, avoid these pitfalls while better aligning with the public interest.

Under current NIH policy, the public access requirement is triggered if any author on a manuscript is supported by NIH funding [1]. This has important implications for trainees and early-career investigators: a K awardee, whose salary support may constitute up to 75% effort, or T32 trainee, can extend this requirement to all work they coauthor, even when the underlying study itself has little or no direct NIH funding. Given that trainee co-authorship is both common and encouraged, this creates a structural tension.

Training and career development awards are explicitly designed to build research experience and publication records, yet they typically provide limited support for direct research costs and little to no support for APCs. In practice, this means early-career investigators write grants to budget for publication fees, negotiate with mentors to cover costs, make strategic publishing decisions based on what they can afford rather than where their work belongs, and spend uncompensated time navigating institutional open access funds, compliance requirements, and deposit procedures, all while performing unpaid peer review for the same journals charging them to publish. As a result, the burden of compliance may fall disproportionately on those with the least financial flexibility, and over time may shape not only who publishes but what kinds of research are feasible to pursue [10–12].

## Reaffirming the original vision

The open access movement, including the founding of PLOS, was grounded in the belief that scientific knowledge is a public good and that its dissemination should serve the broadest possible community [13]. Access was a necessary starting point, but not the sole objective.

As public access becomes the norm, attention should turn to how the system is financed and governed. Alternative publishing models, spanning publisher-driven, funder-driven, institution-driven, and culture-driven reforms (Table 1), demonstrate that the core functions of scholarly communication (evaluation, dissemination, sustainability, and maintenance) can happen outside of per-article payment models. Together, these approaches represent not only technical alternatives but also experiments in governance, redistributing financial responsibility while preserving scientific rigor. Aligning these efforts will be essential to move closer to the original goals of open access: expanding access without introducing new barriers to participation.

**Table 1:** Proposed approaches to address publishing equity in academic medicine.

Model/approach	Description	Limitations and considerations
**Nonprofit** **publishing of publicly funded research** *Publishing model*	Nonprofit publishing venues, including society journals, university presses, and institutionally governed platforms, where revenues are reinvested in dissemination infrastructure rather than extracted as profit [6,13].	Transition is difficult in fields where top venues are commercially owned. Requires coordinated action across societies, scholars, institutions, and funders to shift submission norms.
**Diamond open-access journals** *Publishing model*	No APCs, no subscriptions. Costs are covered by universities, scholarly societies, or public funding [14].	Requires a sustained institutional or society funding commitment. Harder to establish in newer or interdisciplinary fields.
**Overlay journals/preprint servers** *Publishing model*	Decouples peer review from content hosting. Manuscripts posted to medRxiv/bioRxiv are evaluated by an overlay journal at minimal cost.	Preprint culture is less established in clinical medicine than in basic science. Promotion and tenure committees may not yet recognize these venues.
**Institutional repositories** *Institutional*	Author-accepted manuscript deposit provides an APC-free NIH compliance pathway via PubMed Central [2].	Requires institutions to control their own infrastructure. Outsourcing repository operations to commercial vendors preserves extractive revenue even without journal fees.
**Transformative/read-and-publish agreements** *Institutional*	Bundled institutional contracts cover both subscription access and APC costs for authors at signatory institutions, reducing per-article burden for covered investigators.	Does not help investigators at nonsignatory institutions, potentially deepening inequity between well- and under-resourced settings. Has not meaningfully reduced publisher pricing power.
**Institutional policies limiting APC reimbursement** ***Institutional***	Universities decline to cover APCs above a defined threshold or journals without reasonable compliance pathways.	Places a navigational burden on individual investigators who must identify compliant venues. Requires institution-level coordination and clear policy implementation to avoid inconsistent application. Could restrict venue choice.
**Federal purpose license** *Funder policy*	Under standard federal grant terms (2 CFR 200; NIH GPS §8.2.1), the US government holds a royalty-free, irrevocable right to reproduce and publish funded work. NIH asserting this license proactively would protect PMC deposit and provide researchers with leverage against publishers who condition compliance on APC payment [2].	Rarely asserted in practice. Requires NIH policy clarification and enforcement will. Publishers may contest scope.
**Funder APC caps or restrictions** *Funder policy*	NIH sets maximum allowable APCs using funder market power to constrain pricing rather than absorbing costs [9].	Requires coordinated funder policy and enforcement mechanisms. Could restrict venue choice. Risks of creating a market floor effect, reducing price competition.
**Waivers for NIH career development and training awardees**, **and Early-Stage Investigators** *Funder policy*	NIH requires journals receiving APC revenue from established investigators to waive fees for K, T, and F awardees and Early-Stage Investigators as a condition of participation. [10]	Creates a financial disincentive for journals to accept work from fee-waived authors, introducing a structural conflict of interest in peer review if not blinded. Acceptance bias may be subtle and difficult to audit.
**Recognition and compensation for peer review and editorial labor** *Culture/governance*	Makes reviewer and editor contributions visible and valued through financial compensation or formal academic credit, or governance rights over publishing terms [6].	Academic credit for review requires a culture change at the institutional level. Financial compensation could introduce conflicts of interest in editorial decisions. Does not, on its own address APC pricing or market concentration.
**Reform of academic incentive structures** *Culture/governance*	Promotion and tenure criteria that value scientific rigor, reproducibility, and public impact over journal prestige metrics [8].	Requires sustained culture change at departmental, institutional, and national levels. Progress is slow and uneven.

Abbreviations: APC, article processing charge; ESI, early-stage investigator; NIH, National Institutes of Health.

## Conclusions

The NIH’s immediate public access requirement removes a long-standing barrier, ensuring that publicly funded research is no longer locked behind embargoes or paywalls. However, access alone does not guarantee affordability, sustainability, or alignment with the public interest.

As publication costs rise, they risk shaping not only who publishes, but what gets studied, privileging well-funded fields while constraining research in under-resourced areas. In this way, the economics of dissemination can quietly redirect the scientific agenda itself.

Public access was intended to make science available, not to create new barriers at the point of publication. In the NIH 2025 era, that vision and the public access policies meant to advance it, must also ensure that participation in producing and publishing science remains broadly attainable. Without attention to pricing, incentives, and market concentration, public science risks becoming accessible in principle but exclusionary in practice.

## Abbreviations

AAM: author-accepted manuscript
APCs: article processing charges
NIH: National Institutes of Health.
